# The Use of Natural Compounds as a Strategy to Counteract Oxidative Stress in Animal Models of Diabetes Mellitus

**DOI:** 10.3390/ijms22137009

**Published:** 2021-06-29

**Authors:** Marcela Salazar-García, Juan Carlos Corona

**Affiliations:** 1 Laboratorio de Investigación en Biología del Desarrollo y Teratogénesis Experimental, Hospital Infantil de México Federico Gómez, Mexico City 06720, Mexico; 2 Laboratory of Neurosciences, Hospital Infantil de México Federico Gómez, Mexico City 06720, Mexico

**Keywords:** animal model, natural compounds, neuroprotection, oxidative stress, diabetes mellitus

## Abstract

Diabetes mellitus (DM) is a chronic metabolic disease characterised by insulin deficiency, resulting in hyperglycaemia, a characteristic symptom of type 2 diabetes mellitus (DM2). DM substantially affects numerous metabolic pathways, resulting in β-cell dysfunction, insulin resistance, abnormal blood glucose levels, impaired lipid metabolism, inflammatory processes, and excessive oxidative stress. Oxidative stress can affect the body’s normal physiological function and cause numerous cellular and molecular changes, such as mitochondrial dysfunction. Animal models are useful for exploring the cellular and molecular mechanisms of DM and improving novel therapeutics for their safe use in human beings. Due to their health benefits, there is significant interest in a wide range of natural compounds that can act as naturally occurring anti-diabetic compounds. Due to rodent models’ relatively similar physiology to humans and ease of handling and housing, they are widely used as pre-clinical models for studying several metabolic disorders. In this review, we analyse the currently available rodent animal models of DM and their advantages and disadvantages and highlight the potential anti-oxidative effects of natural compounds and their mechanisms of action.

## 1. Introduction

Diabetes mellitus (DM) is a chronic metabolic disorder characterised by insulin deficiency (pancreatic β-cell dysfunction) and insulin resistance. There are three widely accepted major forms of DM, including gestational DM, type 1 diabetes mellitus (DM1), and type 2 diabetes mellitus (DM2), which accounts for approximately 90% of all cases of DM. DM2 is associated with several factors, including chronic hyperglycaemia, hyperlipidaemia, and hypertension, as a result of insulin resistance or insulin deficiency [1,2]. The global prevalence of diabetes in 2019 was estimated at 9.3% (463 million patients), which is expected to increase to 10.2% by 2030 (578 million) and 10.9% by 2045 (700 million) [3]. Normally, insulin lowers blood glucose levels by stimulating peripheral glucose uptake and suppressing hepatic glucose production; however, a dysfunction could conduct to insulin resistance. Thus, an increase in insulin secretion or β-cell mass can compensate for the insulin resistance by normal β cells, producing more circulating insulin; however, insufficient compensation results in the onset of glucose intolerance, leading to exacerbation of hyperglycaemia. Once exhausted, pancreatic β cells can no longer overproduce insulin and DM develops [2,4,5].

## 2. Oxidative Stress in Diabetes Mellitus

Prolonged hyperglycaemia and hyperlipidaemia in DM can lead to oxidative stress as the result of reactive oxygen species (ROS) overproduction in the cytosol or mitochondria, which counteracts the cellular redox balance and induces more oxidative stress [6,7,8]. Oxidative stress, therefore, causes significant damage to various cellular biomolecules, including proteins, lipids, and DNA [9,10]. The resulting dysregulated expression in numerous genes and proteins leads to impaired insulin secretion and signalling [11], which contributes to the development and progression of DM complications such as retinopathy, nephropathy, non-alcoholic fatty liver disease, hypertension, and cardiovascular diseases, jointly known as metabolic syndrome [12,13,14,15]. DM is also associated with a long list of risk factors, including gestational DM, sedentary lifestyle, genetics, and obesity [5,16].

As pancreatic β-cell function gradually worsens, hyperglycaemia becomes apparent. Glucose toxicity in β cells in DM is, therefore, a significant cause of oxidative stress [9,17,18]. Furthermore, the expression of antioxidant enzymes such as superoxide dismutase (SOD), catalase (CAT), and glutathione peroxidase (GPx) is low in pancreatic β cells; consequently, β cells could be a target of oxidative stress-mediated tissue damage [18,19,20]. High glucose exposure increases oxidative stress in human islets and pancreatic β-cell lines, and GPx overexpression increases GPx activity and protects islets against adverse effects [21]. Oxidative stress is therefore a feature that contributes to insulin resistance and β-cell dysfunction in DM. The diabetic brain can also be injured, showing a wide profile of microstructural and macrostructural changes, leading to neurovascular deterioration, progressive cognition dysfunction, excessive oxidative stress, and neurodegeneration [22].

## 3. Animal Models of Diabetes Mellitus

Due to their relatively similar physiology to humans and ease of handling and housing, mice and rats are widely used as pre-clinical animal models for studying metabolic disorders [23,24]. These rodents are therefore useful as models for investigating DM-related molecular and cellular events and can provide knowledge about the effects of anti-DM agents in individual or groups of tissues.

Streptozotocin (STZ) is an antibiotic that produces pancreatic islet β-cell destruction and is widely used experimentally to produce a DM1 model. Within 48 h, a single high dose of STZ causes complete β-cell necrosis and DM [25]. However, low doses of STZ for 5 days elicit partial β-cell loss, which results in hypo-insulinaemia and hyperglycaemia [26,27]. There are data indicating a major role for oxidative stress as a consequence of STZ-induced DM in rats, which demonstrated increased oxidative stress in the early stages of STZ-induced DM in rats as well as mitochondrial dysfunction [28]. In addition, STZ-induced DM in female rats on day 5 of pregnancy affected the intra-uterine developmental timeline, which resulted from maternal DM and preceded embryo implantation, restricted embryo–foetal growth, and delayed the maturation and remodelling of the structures derived from neural crest cells [29]. The administration of STZ-nicotinamide (NA) in rats has been proposed to induce DM2, because STZ causes pancreatic β-cell damage, whereas NA in rats partially protects pancreatic β cells against STZ [30].

Food highly enriched in fats, commonly known as high-fat diets (HFDs), either alone or in combination with sodium chloride or glucose is considered to mimic human DM2 [23]. There are data indicating the role of oxidative stress in HFDs, given that ROS production might be an initial event triggering HFD-induced insulin resistance [31]. To induce DM2 in a shorter period, HFD combined with low-dose STZ has been employed [32].

The C57BL/6J mouse strain is extensively used as a model for diet-induced obesity (DIO) due to susceptibility to developing severe obesity, glucose intolerance, elevated adiposity, and moderate insulin resistance [33,34]. However, the DIO C57BL/6J model is not the perfect choice for studying the effects of diabetes because this model rarely develops hyperglycaemia or islet atrophy when fed an HFD [23,35].

Alloxan is an organic compound, urea derivative, carcinogen, and cytotoxic glucose analogue and is, therefore, one of the most commonly employed diabetogenic agents. Alloxan promotes selective pancreatic β-cell necrosis by promoting ROS accumulation [36,37].

Certain genetic rodent models have been extensively employed as pre-clinical models for DM, such as leptin-null (*ob/ob*) mice and leptin receptor (*db/db*) mutant mice [38,39]. O*b/ob* mice maintained on a C57BL/6J genetic background exhibit early-onset severe obesity, reduced energy expenditure, hyperinsulinaemia, insulin resistance, and mild hyperglycaemia [40]. In contrast, the *ob/ob* mice maintained on a C57BLKS/J background exhibit pronounced DM, severe hyperglycaemia, and pancreatic islet atrophy, leading to premature death [41]. D*b/db* mice are maintained on a C57BLKS/J genetic background, which imparts the phenotypic differences of severe diabetes [41]. There are data pointing to oxidative stress as a contributor to the patho-physiologic disorders observed in *ob/ob* mice, wherein an increase in oxidative stress has been observed in *ob/ob* mice as part of the DM-associated disorders [42,43]. Oxidative damage has been demonstrated in the brains of DM2 *db/db* mice [44].

There is a sex difference in most rodent DM2 models, with the Zucker Diabetic Fatty (ZDF) rat providing one example. Female ZDF rats maintain normal insulin and glucose levels throughout their lives, despite developing obesity to a similar degree as the males. In contrast, male ZDF rats develop obesity, insulin resistance, severe hyperglycaemia, and hyperlipidaemia as a consequence of DM2 [45,46]. Therefore, the sexual dimorphism in blood glucose can be translated to human beings. The role of oxidative stress can also be a contributor to the pathophysiologic disorders observed in ZDF rats because increased ROS production and mitochondrial dysfunction has been shown in ZDF rat brains [47].

Otsuka Long-Evans Tokushima Fatty (OLETF) rats develop DM and lack the cholecystokinin receptor type A, a gut-derived peptide hormone that works as a peripheral satiation signal. Due to β-cell collapse, the characteristic symptoms are polydipsia and polyuria [48,49]. Evidence also points to the role of oxidative stress as a consequence of DM in OLETF rats because increased oxidative stress levels have been shown in the plasma, pancreas, and liver of OLETF rats as well as significantly decreased plasma SOD-like activity [50].

Goto–Kakizaki (GK) rats have neonatal β-cell mass deficiency, with 50% depletion in adult rats. Defects in β-cell metabolism and function are elicited, and a loss of β-cell mass occurs with chronic hyperglycaemia, oxidative stress, and inflammation. GK rats therefore constitute a non-obese polygenic DM2 model [51,52,53].

## 4. Natural Compounds against Oxidative Stress in Animal Models of Diabetes Mellitus

The use of natural compounds in animal DM models has been shown to improve glycaemic control, reduce inflammation, decrease oxidative stress and neurodegeneration, and prevent various complications of DM [54,55,56,57]. Table 1 summarises the outcomes for the protective effects of natural compounds against oxidative stress in animal DM models.

### 4.1. Natural Polyphenols

Natural polyphenols are secondary metabolites of plants and found largely in fruits; vegetables; cereals; and beverages such as wine, coffee, tea, and beer. Polyphenols are, therefore, considered the most abundant antioxidantantioxidants in the human diet, and diets rich in polyphenols provide protective effects against DM, cardiovascular diseases, cancer, and several neurodegenerative diseases. More than 8000 polyphenols have been identified in several plants and have been classified into various classes including phenolic acids, flavonoids, stilbenes, and lignans [58,59]. The effects of diverse natural polyphenols against oxidative stress have been demonstrated in various rodent DM models. Resveratrol is a natural polyphenol compound, widely found in grapes and blueberries. Long-term treatment with resveratrol improved neuronal injury and cognitive performance in STZ-induced diabetic rats by attenuating inflammation, increasing malondialdehyde (MDA) levels (a marker of lipid peroxidation), and increasing SOD, CAT, and glutathione (GSH) levels in the hippocampus [60]. In STZ-induced diabetic rats, resveratrol treatment partially normalised oxidative biomarkers, such as the total oxidant status (TOS) and MDA levels, and improved CAT and SOD1 mRNA levels. Similarly, CAT, GPx, and glutathione S-transferase (GST) activity was also increased in the brains of treated diabetic rats [61]. The administration of resveratrol in STZ-NA-induced DM2 rats decreased blood glucose and glycated haemoglobin (HbA1c) levels and increased the antioxidant activity of SOD, CAT, GPx, and GSH in the liver. Resveratrol has also been shown to increase the expression of peroxisome proliferator-activated receptor-gamma (PPARγ) and fatty aldehyde dehydrogenase (FALDH) genes in adipose tissue. PPARγ is a transcription factor involved in adipogenesis, regulation of adipocyte gene expression, and glucose metabolism and acts as a key energy balance regulator. PPARγ activation also increases antioxidant defence and regulates mitochondrial function [62]. FALDH is an enzyme that can metabolise 4-hydroxynonenal (HNE) and decrease HNE-induced ROS production [63].

**Table 1:** The use of natural compounds as a strategy for counteracting oxidative stress in animal DM models.

Compound	Model	Outcome		Reference
Polyphenols		Oxidative Markers and Protection	Antioxidant Proteins	
Resveratrol	STZ-induced DM rats and STZ-NA-induced DM rats	Improved neuronal injury and cognitive performance by attenuating inflammation, ↓ MDA levels. ↓ TOS and MDA levels. ↓ blood glucose and HbA1c levels and ↑ PPARγ and FALDH genes.	↑ SOD, CAT, and GSH levels in the hippocampus. Improved SOD1 and CAT mRNA levels, ↑ CAT, GPx, and GST activity in the brain. ↑ SOD, CAT, GPx, and GSH activity in the liver	[60,61,63]
Curcumin	STZ-induced DM rats	↓ blood glucose, ↑ neuroprotection. ↓ TBARS levels, ↑ AGE, AGE-R1 receptor, glyoxalase-1 in the kidneys and liver and prevented dyslipidaemia.	↑ SOD, CAT, GPx, and GSH activity in the hippocampus. ↑ SOD, CAT, and PON1 activity.	[64,65]
Syringic acid	STZ-induced DM rats	↓ blood glucose, improved memory, learning and movement deficiency, ↓ MDA levels in the brain, sciatic nerve and spinal cord, ↑ mRNA expression of PGC1α and NRF1 in the brain.		[66]
**Flavonoids**				
Quercetin	STZ-induced DM rats, STZ-NA-induced DM2 rats, HFD/STZ-induced DM2 rats, and *db/db* DM2 mice	↓ blood glucose, ↓ plasma TBARS, and hydroperoxides. ↓ MDA levels in erythrocytes, ↓ serum NO levels. ↓ MDA levels. ↓ AOPP and MDA levels. ↓ ER-stress and MDA levels. Ameliorated neurodegeneration, improved learning and memory impairment, ↓ MDA levels. ↓ MDA levels, ↑ ATP generation and improved changes in mitochondria, ↑ AMPK, SIRT1, PGC1α, TFAM, and NRF1 in plasma and sciatic nerves.	↑ SOD and CAT activity, ↑ vitamin C and E levels in erythrocytes and plasma. ↑ SOD, CAT, and GPx levels in pancreatic tissue. ↑ SOD, CAT, and GPx activity and ↑ SOD1, CAT, and GPx1 protein levels in the heart. ↑ GSH levels in pancreatic tissue. ↑ SOD, CAT, and GPx activities in the pancreas. ↑ SOD, CAT, and GPx activity in the brain.	[67,68,69,70,71,72,73]
Kaempferol	STZ-induced DM rats and STZ-induced DM mice	↓ blood glucose, ↓ TBARS, and hydroperoxides. ↓ DHE level and 3-nitrotyrosine, ↑ Nrf-2, and NQO1 expression levels.	↑ SOD, CAT, GPx, and GST activity, ↑ GSH, vitamin C, and vitamin E in the plasma, heart, liver, and kidneys.	[74,75]
Luteolin	STZ-induced DM rats	Improved neuronal injury and cognitive performance, ↓ MDA levels.	↑ SOD, CAT, and GSH activity in the cerebral cortex and hippocampus.	[76]
Ficus deltoidea	STZ-induced DM rats and STZ-NA-induced DM2 rats	Improved spatial learning and memory, ↓ TBARS in the brain. ↓ blood glucose and ↓ MDA levels in the pancreas and liver.	↑ SOD and GPx activity in the brain. ↑ SOD, CAT, GPx, and GSH levels in the pancreas and liver.	[77,78]
Chrysin	STZ-induced DM rats and HFD/sucrose-induced DM2 rats	↓ blood glucose, improved learning, and memory, ↓ MDA levels in the brain. ↓ blood glucose and lipids and ↑ insulin, ↓ MDA levels, ↓ OH and H_2_O_2_ in the gastrocnemius muscle.	↑ SOD, CAT, and GSH activity in the cerebral cortex and hippocampus.	[79,80]
**Propolis**				
Chinese	Alloxan-induced DM rats and STZ-induced DM rats	↓ blood glucose, ↓ MDA, NO, and NOS. ↓ blood glucose, ↓ HbA1c, ↓ MDA, ↓ ROS and RNS in serum.	↑ SOD levels in blood.	[81,82]
Chinese and Brazilian	STZ-induced DM rats	↓ blood glucose, ↓ MDA levels in blood and kidneys.	↑ SOD in blood, ↑ CAT in kidneys, and ↑ GPx in the liver.	[83]
Croatian	Alloxan-induced DM mice	↓ MDA levels in liver and ↑ antiradical activity and ↓ β-carotene degradation.		[84]
Malaysian	STZ-induced DM rats	↓ blood glucose, ↑ TAC and ↓ MDA in the pancreas.	↑ SOD, CAT, GPx, GR, and GST activities.	[85]
Taiwanese	HFD/STZ-induced DM2 rats	↓ blood glucose and ↓ TBARS in serum.	↑ SOD and GPx activities.	[86]
Mexican	STZ-induced DM mice	↓ blood glucose and ↑ plasma insulin levels.	↑ SOD, CAT, and GPx activities in the pancreas.	[87]
CAPE	STZ-induced DM rats	↑ HO-1 and GGCL, ↓ nitrite/nitrate levels, and ↓ protein expression of iNOS in the pancreas.		[88]
**Alkaloids**				
Berberine	HFD/STZ-induced DM2 rats, STZ-NA-induced DM2 mice, and HFD/glucose-induced DM2 hamsters	↓ MDA levels. ↓ blood glucose, and ↓ MDA levels in the liver and brain. ↓ MDA plasma levels, ↓ TBARS, and ↓ blood glucose, improved memory impairment, axonopathy, and tau hyperphosphorylation.	↑ SOD, CAT, GPx, and GSH activity in the liver and serum. ↑ SOD1 mRNA in liver, ↑ SOD and CAT activities in the kidneys. ↑ SOD activity in plasma.	[89,90,91,92]
Vindoline	STZ/fructose-induced DM2 rats	↓ blood glucose and↑ ORAC. ↑ FRAP in the cardiac tissue, ↑ ORAC, ↓ MDA levels in the kidneys.	↑ SOD activity in the liver. ↑ SOD activity.	[93,94]
Oxymatrine	HFD/STZ-induced DM2 rats	↓ blood glucose and ↓ MDA levels.	↑ SOD, CAT, and GPx activity in the kidneys.	[95]
**Ginseng**				
Ginsenosides	GK DM2 rats	↓ blood glucose, improved learning, and memory decline, ↓ MDA levels.	↑ SOD activity in the hippocampus.	[96]
Korean red	OLETF DM rats	↓ blood glucose and ↓ MDA levels.	↑ GPx activity in plasma.	[97]

Curcumin is a polyphenolic compound that has been shown to have anti-hyperglycaemic, anti-inflammatory, and antioxidant activity, attenuating DM complications [98]. Treatment with curcumin had a neuroprotective effect in the hippocampus of STZ-induced diabetic rats and significantly increased the activity of SOD, CAT, GPx, and GSH [64]. In addition, curcumin treatment of STZ-induced diabetic rats decreased the plasma levels of the oxidative biomarker thiobarbituric acid reactive substances (TBARS); increased the activity of the antioxidant enzymes SOD, CAT, and paraoxonase-1 (PON1) in the kidneys and liver; and increased the levels of advanced glycation end product (AGE) and detoxification system components (AGE-R1 receptor and glyoxalase-1). Additionally, the combination of curcumin and aminoguanidine, a therapeutic agent with anti-AGE activity, prevented dyslipidaemia in diabetic rats [65]. The neuroprotective effects of syringic acid, a natural polyphenolic derivative of benzoic acid, were evaluated in STZ-induced diabetic rats. Treatment with a syringic acid significantly improved the rats´ memory; improved their learning and movement deficiency; reduced MDA levels in the brain, sciatic nerve, and spinal cord, and upregulated the brain mRNA expression of PPARγ coactivator 1 alpha (PGC1α) and nuclear respiratory factor (NRF1) [66]. PGC1α is a transcription coactivator that regulates mitochondrial biogenesis and induces NRF1 transcription [99]

### 4.2. Flavonoids

Flavonoids belong to a large group of natural polyphenolic phytochemicals with several subclasses and have shown beneficial effects, such as antioxidantantioxidant activity in several neurodegenerative and neuropsychiatric disorders, as well as anti-inflammatory, anti-obesity, anti-diabetic, and cardioprotective activity. Quercetin is a flavonoid compound present in a wide variety of fruits and vegetables and has been shown to exert therapeutic effects [100]. The oral administration of quercetin to STZ-induced diabetic rats resulted in a decrease in blood glucose, plasma TBARS, and hydroperoxides levels. Quercetin also increased SOD and CAT activity and vitamin C and E levels to near normal values in the erythrocytes and plasma of treated rats [67]. Furthermore, quercetin treatment decreased MDA levels in erythrocytes and serum nitric oxide (NO) and increased SOD, CAT, and GPx levels in the pancreatic tissue of STZ-treated rats [68]. Quercetin treatment of STZ-NA-induced DM2 rats decreased MDA levels and increased SOD, CAT, and GPx activity levels and caused a significant increase in SOD1, CAT, and GPx1 protein levels in the heart tissue homogenates of treated rats [69]. Similarly, the administration of quercetin resulted in a significant decrease in advanced oxidation protein products (AOPP) and MDA levels along with a significant increase in GSH levels in pancreatic tissue homogenates of HFD/STZ-induced DM2 rats [70]. Similarly, quercetin treatment reduced ER stress-mediated oxidative stress, reduced MDA levels, and improved SOD, CAT, and GPx activity in the pancreas of STZ-induced diabetic rats [71]. Moreover, quercetin ameliorated neurodegeneration; improved learning and memory impairment; reduced MDA levels; and increased SOD, CAT, and GPx activity in the brain of db/db mice, which is an animal model of DM2 [72]. Lastly, quercetin decreased MDA levels, increased ATP generation, and corrected morphological changes in mitochondria in the plasma and sciatic nerves of STZ-induced diabetic rats. Quercetin treatment also promoted the expression of phosphorylated adenosine 5’-monophosphate-activated protein kinase (AMPK), sirtuin 1 (SIRT1) a sensor of energetic metabolism, PGC1α, TFAM, and NRF1 (key regulators of mitochondrial biogenesis, energy metabolism, and oxidative phosphorylation) [73]. Kaempferol is a flavonoid that naturally occurs in a variety of vegetables, fruits, tea, and wine and exhibits a wide range of kinds of pharmacological activity, including antioxidant, anti-inflammatory, neuroprotective, and anti-diabetic activity [101]. The ameliorative effects of kaempferol have been observed in STZ-induced diabetic rats, with a significant decline in TBARS and lipid hydroperoxide towards normal levels; increased activity of SOD, CAT, GPx, and GST; and increased levels of non-enzymatic antioxidants towards normal levels, such as GSH, vitamin C, and vitamin E in the plasma, heart, liver, and kidneys of treated rats [74]. Kaempferol in the heart tissue of STZ-induced diabetic mice decreased the generation of oxidative stress measured with dihydroethidium (DHE), reduced the generation of 3-nitrotyrosine (a product of peroxynitrite), increased the expression of the nuclear factor erythroid 2-related factor 2 (Nrf-2) signalling pathway, which plays a major role in regulating of oxidative stress and increased NQO1 expression. In vitro, kaempferol decreased the levels of MDA and DHE and enhanced SOD activity in H9c2 cells treated with high quantities of glucose [75].

Luteolin is a flavonoid with antioxidant and neuroprotective activity. Long-term treatment with luteolin improved neuronal injury and cognitive performance in STZ-induced diabetic rats; significantly inhibited the increase in MDA levels; and improved SOD, CAT, and GSH activity in the cerebral cortex and hippocampus [76]. The leaves of Ficus deltoidea have been extensively used as herbal medicine to normalise blood sugar levels and contain more than 20 varieties of flavonoids, giving the leaves antioxidant effects [102]. Treatment with Ficus deltoidea in STZ-induced diabetic rats improved spatial learning and memory, increased SOD and GPx activity, and significantly reduced TBARS in the brains of diabetic rats [77]. Ficus deltoidea also significantly increased SOD, CAT, GPx, and GSH levels and reduced MDA levels in the pancreas and livers of STZ-NA-induced DM2 rats [78]. Long-term treatment with the flavonoid chrysin suppressed the increase in MDA content in the brains of STZ-induced diabetic rats; increased the activity of SOD, CAT, and GSH in the cerebral cortex and hippocampus; and improved learning and memory function [79]. Treatment with chrysin normalised the altered blood glucose levels, serum insulin levels, and lipid profile and significantly reduced the levels of MDA, hydroxyl radical (OH), and hydrogen peroxide (H_2_O_2_) in the gastrocnemius muscle of HFD/sucrose-induced DM2 rats [80].

### 4.3. Propolis

**Figure 1:**
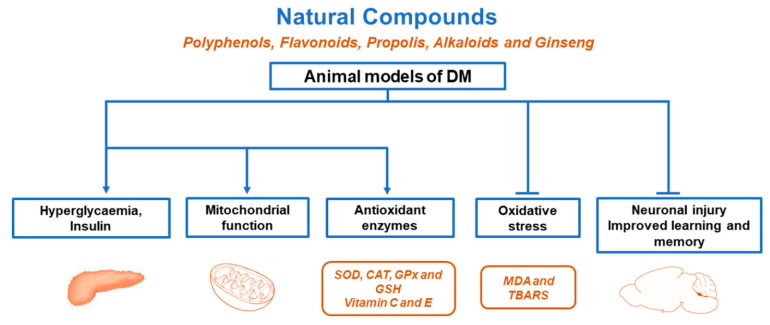
Protective effects of various natural compounds in animal DM models. Natural compounds prevented hyperglycaemia, ROS production, and neuronal injury; increased insulin secretion and antioxidant enzymes; and improved mitochondrial function, learning, memory, and lipid metabolism, among others.

Propolis is a complex resinous material that consists of sap, bark, and bee excreta accumulated in beehives. More than 300 compounds including flavonoids, phenolic acids, volatile organic compounds, phenolic aldehydes, alcohols, ketones, sesquiterpenes, quinones, coumarins, steroids, and amino acids have been isolated from propolis [103]. The effects of propolis against oxidative stress have been demonstrated in diverse rodent DM models. Extracts of propolis from north China have significantly decreased levels of blood glucose, MDA, NO, and nitric oxide (NOS) synthetase, whereas blood SOD levels were increased in alloxan-induced rats [81]. In STZ-induced diabetic rats, Chinese and Brazilian propolis significantly inhibited body weight loss and blood glucose increases; decreased MDA levels in the blood and kidneys; and slightly increased SOD in the blood, CAT in the kidneys, and GPx in the liver [83]. Treatment with Croatian propolis preparations prevented bodyweight reduction in alloxan-induced diabetic mice and significantly decreased MDA concentrations in the liver. Moreover, 2,2-diphenyl-1-picrylhydrazyl (DPPH) is frequently used to determine radical-scavenging activity, and propolis preparations have demonstrated anti-radical activity. The antioxidant capacity of propolis preparations has been evaluated using a β-carotene–linoleic acid assay, showing that they inhibited β-carotene degradation [84]. Malaysian propolis increased the activity of SOD, CAT, GPx, glutathione reductase (GR), and GST; total antioxidant capacity (TAC) and MDA level was significantly decreased in the pancreas of STZ-induced diabetic rats [85]. Taiwanese green propolis increased SOD and GPx activity and produced a decrease in a TBARS assay in the serum of HFD/STZ-induced DM2 rats [86]. Similarly, Mexican propolis significantly inhibited increases in blood glucose and loss of body weight in STZ-induced mice, increased plasma insulin levels, and increased the activity of SOD, CAT, and GPx enzymes in the pancreas. The chemical composition analysis showed that Mexican propolis was rich in flavonoids such as kaempferol, quercetin naringin, pinocembrin, naringenin, acacetin, chrysin, and luteolin [87]. Moreover, extract of Chinese propolis decreased the levels of HbA1c, which also resulted in the decrease of MDA, ROS, and reactive nitrogen species (RNS) in the serum of STZ-induced diabetic rats [82]. The protective effects of caffeic acid phenethyl ester (CAPE), a natural phenolic compound derived from honeybee hive propolis, were demonstrated in STZ-induced diabetic rats. The expression levels of antioxidant enzyme-related proteins, such as heme oxygenase-1 (HO-1) and gamma-glutamylcysteine ligase (GGCL), were restored. Nrf-2 modulates the expression of genes such as HO-1 and GGCL. CAPE also decreased the nitrite/nitrate levels and decreased the protein expression of iNOS in the pancreas of STZ-induced diabetic rats [88]. Figure 1 summarises the protective effects of various natural compounds in animal DM models.

### 4.4. Alkaloids

Alkaloids are a class of natural compounds derived from natural sources, such as plants. There are approximately 20,000 known alkaloids, most of which have been isolated from plants. However, alkaloids have also been found in algae; marine organisms; and animals such as insects, toads, and salamanders [104]. Berberine, an isoquinoline alkaloid derived from the ancient Chinese herb Coptis Chinensis French, has been used to treat DM for thousands of years, with a broad range of effects, such as decreased oxidative stress, reduced inflammation, and protection against neurodegenerative diseases [105]. Berberine decreased MDA levels and increased SOD, CAT, GPx, and GSH activity in the livers and sera of HFD/STZ-induced DM2 rats [89]. Berberine also increased hepatic SOD1 mRNA expression and kidney SOD and CAT activity to normal levels and decreased MDA levels in the livers and brains of STZ-NA-induced DM2 mice [90]. In hamsters fed a high content of glucose and HFD, treatment with berberine reduced the plasma levels of MDA and TBARS and increased plasma SOD activity [91]. Berberine significantly attenuated memory impairment, axonopathy, and tau hyperphosphorylation in HFD/STZ-induced DM2 rats. Primary neurons treated simultaneously with high quantities of glucose and berberine improved axonal transport, decreased MDA levels, and increased SOD activity [92]. Vindoline, an indole alkaloid present in the leaves of the Catharanthus roseus plant, might serve as an insulin sensitiser. The administration of vindoline significantly improved oxygen radical absorbance capacity (ORAC), an assay measuring the ability of antioxidants in a particular sample to scavenge radicals, and improved SOD activity in the livers of rats exposed to 10% fructose with STZ employed as a DM2 model [94]. Using the above rat DM2 model, treatment with vindoline improved the ferric-reducing antioxidant power (FRAP) in cardiac tissue significantly improved the ORAC and SOD activity and significantly reduced MDA levels in the kidneys [93]. Oxymatrine, a major quinolizidine alkaloid in Sophora flavescens, exhibits several pharmacological effects, such as anti-inflammatory, anti-oxidative, and neuroprotective activity. Treatment with oxymatrine effectively increased the activity of SOD, CAT, and GPx and decreased MDA content in the kidneys of HFD/STZ-induced DM2 rats [95].

### 4.5. Ginseng

Ginseng is a traditional Chinese herb containing active ingredients known as ginsenosides, the main compounds that elicit the therapeutic actions of ginseng. Ginsenoside treatment significantly decreased MDA activity and increased SOD activity in the hippocampus and improved learning and memory decline in the GK DM2 rat model [96]. Korean red ginseng has antioxidant and cardio-protective effects, and therefore, treatment with this herb decreased MDA levels and increased GPx activity in the plasma of DM2 OLETF rats [97].

## 5. Clinical Trials

Regardless of the great attributes and potentials of natural compounds used for the management or treatment of DM in rodent models, there is limited information concerning their efficacy in human clinical trials. Hence, only a few natural compounds have been explored in human clinical trials. Some natural components that have been studied in clinical trials are as follows. The antidiabetic activity of ginseng has been demonstrated in clinical trials [106]. Grape polyphenols prevented oxidative stress and insulin resistance in first-degree relatives of DM2 patients [107]. A moderate antidiabetic effect of cinnamon was observed in a randomised placebo-controlled clinical trial [108]. Allium cepa L., also known as the bulb onion or common onion, has been shown to exhibit antidiabetic activity [109]. Aloe vera extracts have been shown to exhibit a substantial decrease in glucose [110]. Finally, it has been shown that some natural compounds could improve DM in clinical trials. However, more clinical trials and prospective, well-designed research are still needed to confirm these results.

## 6. Conclusions

As a chronic metabolic disease with several causative factors, DM is characterised by insulin deficiency and insulin resistance. In DM2, hyperglycaemia and hyperlipidaemia generate oxidative stress, causing cellular metabolic dysregulation. We focused on DM rodent models that play an important role in presenting the pathogenesis of human DM and its complications. Accordingly, diabetic rodent models are essential for studying several complications of DM, such as oxidative stress, and for developing new therapeutic strategies and novel drugs. The use of natural compounds for the management and treatment of DM is increasing, because the available medications sold over the counter are very expensive and have several side effects. The study of natural compounds with anti-diabetic potential is gaining greater attention daily because these compounds possess the ability to mitigate DM via several mechanisms such as the regulation of hyperglycaemia, decrease of oxidative stress, and neuronal injury; increased insulin secretion and antioxidant enzymes; and improved mitochondrial function, learning, memory, and lipid metabolism in rodent models. Thus, natural compounds could contribute to expanding the therapeutic options for treating or reducing the complications associated with DM. Nevertheless, only a few natural compounds have been used in clinical studies. Hence, there is a need for further research progress in the area of natural compounds with anti-diabetes activity and characterised as potential compounds employed as clinical medication or dietary supplements to ameliorate the management or treatment of DM.

## Data Availability

Not applicable.
